# BARD1-mediated stabilization of METTL14 promotes retinal neovascularization by m6A-modifying MXD1 mRNA on a YTHDF2-dependent manner

**DOI:** 10.7150/thno.110122

**Published:** 2025-04-13

**Authors:** Xianyang Liu, Shuhao Zeng, Jiayu Meng, Qian Zhou, Fan Cao, Baorui Chu, Chao Wu, Yakun Wang, Hui Feng, Xiaorui Bi, Xinyuan Chen, Wenxian Yang, Meng Tian, Hui Yang, Ke Hu, Shengping Hou

**Affiliations:** 1 The First Affiliated Hospital of Chongqing Medical University, China.; 2 Beijing Institute of Ophthalmology, Beijing Tongren Eye Center, Beijing Tongren Hospital, Capital Medical University, Beijing Ophthalmology & Visual Sciences Key Laboratory, Beijing, 100730, China.; 3 Sichuan Provincial Key Laboratory for Human Disease Gene Study, Sichuan Provincial People's Hospital, University of Electronic Science and Technology of China, Chengdu 611731, China.; 4 Department of Ophthalmology, Qilu Hospital, Cheeloo College of Medicine, Shandong University, Jinan, China.

**Keywords:** retinal angiogenesis, microglia, m6A, ubiquitination

## Abstract

Retinal vascular diseases are typified by the proliferation of irregular and leaky microvessels, resulting in vision impairment. Although the etiology of retinal angiogenesis is not yet fully understood, it is evident that microglia play a pivotal role in promoting angiogenesis.

**Methods:** In vivo, the METTL14 conditional knockout (cKO) mouse was constructed to investigate the role of METTL14 in oxygen-induced retinopathy (OIR). In vitro, a combination of methylated RNA immunoprecipitation sequencing (MeRIP-seq), RNA-sequencing (RNA-seq), RNA Immunoprecipitation (RIP) assay, dual-luciferase reporter assays, and Chromatin immunoprecipitation-qPCR (ChIP-qPCR), was performed to explore the underlying mechanisms.

**Results:** The proteomic analysis of hypoxic microglia has uncovered a pronounced enrichment in pathways related to RNA modification. Western blot has revealed that N6-methyladenosine (m6A) methyltransferase-like 14 (METTL14) exhibits the most significant increase among the RNA methylases. METTL14 cKO mice within an OIR model showed fewer neovascular formations. Additionally, in co-culture with sh-METTL14 HMC3 cells, HRMECs also exhibited reduced angiogenesis capabilities. Mechanically, E3 ubiquitin-protein ligase BARD1 can directly interact with METTL14, leading to an up-regulation of METTL14 protein level in hypoxic microglia. METTL14 could directly modifies and regulates the transcription factor MAX Dimerization Protein 1 (MXD1), which is subsequently recognized by the m6A "reader" YTH domain-containing family protein 2 (YTHDF2). Consequently, the modified MXD1 modulates the expression of VEGFA and VCAM1, promotes retinal neovascularization.

**Conclusion:** Our study highlights the critical role of METTL14 in the OIR model and suggests a novel therapeutic target for addressing retinal vascular diseases.

## Introduction

Retinal vascular diseases, encompassing conditions such as retinopathy of prematurity (ROP), diabetic retinopathy (DR), and age-related macular degeneration (AMD), are typified by the proliferation of irregular and leaky microvessels 1-4. These vascular anomalies are a frequent cause of severe vision loss. The use of anti-vascular endothelial growth factor (VEGF) therapies has become a standard practice in the clinical management of retinal neovascularization 5, 6. Nonetheless, these treatments exhibit a limited efficacy, generally achieving only a partial blockage of angiogenesis. Therefore, alternative therapeutic modalities are urgently needed.

Microglia, the resident immune cells of the central nervous system (CNS), not only serve in immune surveillance but also are key players in vascular development and remodeling under pathological states within the CNS 7-9. The previous study has shown that depletion of retinal microglia via intravitreal clodronate liposome administration leads to reduced retinal vascular density, highlighting the significance of microglia in angiogenesis 10.

Microglia exhibit high plasticity and undergo reprogramming through epigenetic modification 11-13. Particularly noteworthy among these modifications is N6-methyladenosine (m6A) methylation, considered one of the most crucial epigenetics alterations 14, 15. This modification, the most prevalent among eukaryotic mRNA base modifications, is found across a wide spectrum of transcripts 16-19. Influencing RNA maturation, export, translation, and decay, m6A methylation relies on the dynamic interactions of the "writers," "erasers," and "readers" of the m6A marks, serving as a central mediator in cellular responses to emergencies such as hypoxia and inflammation 20-22. Evidence suggests that methyltransferase-like 3 (METTL3) promotes microglia inflammation by regulating MEF2C mRNA m6A modification induced by lipopolysaccharide (LPS) treatment 23. Moreover, Li et al. 24 found that depletion of methyltransferase-like 14 (METTL14) alleviated MCAO-induced brain injury, likely by shifting microglia/macrophage polarization from M1 to M2 and restraining the NLRP3 inflammasome axis in microglia. Furthermore, targeting the m6A mRNA demethylase fat mass and obesity-associated (FTO) suppresses the release of vascular endothelial growth factor and choroidal neovascularization 25. These results suggest that m6A modification plays an important role in microglia function and maybe involved into microglia-mediated angiogenesis.

In our study, aimed at elucidating the crucial biological process underlying retinal angiogenesis, we reanalyzed proteomic data from our previous studies on hypoxic HMC3 cells. Clusters of Orthologous Groups (COG) analysis revealed a notable enrichment in transcription and RNA processing and modification. Subsequently, we identified a significant increase in m6A "Writer" METTL14 in vivo and in vitro. This increase was associated with a direct interaction between METTL14 and the E3 ubiquitin-protein ligase BARD1, which in turn resulted in an elevated level of METTL14 protein. Furthermore, METTL14 conditional knockout (cKO) mice within an oxygen-induced retinopathy (OIR) model showed fewer neovascular formations. To delve deeper into the underlying mechanisms, we employed a combination of MeRIP-seq, RNA-seq, RIP assay, dual-luciferase reporter assays, and ChIP-qPCR, revealing METTL14 directly modifies and regulates the mRNA of MAX Dimerization Protein 1 (MXD1). The modified mRNA was specifically recognized by the "reader" protein YTH domain-containing family protein 2 (YTHDF2). Consequently, our findings not only elucidate the pivotal role of METTL14 in the pathogenesis of OIR but also suggest its potential as a novel therapeutic target for treating ROP.

## Results

### The protein level of METTL14 is significantly increased in OIR

**Figure 1:**
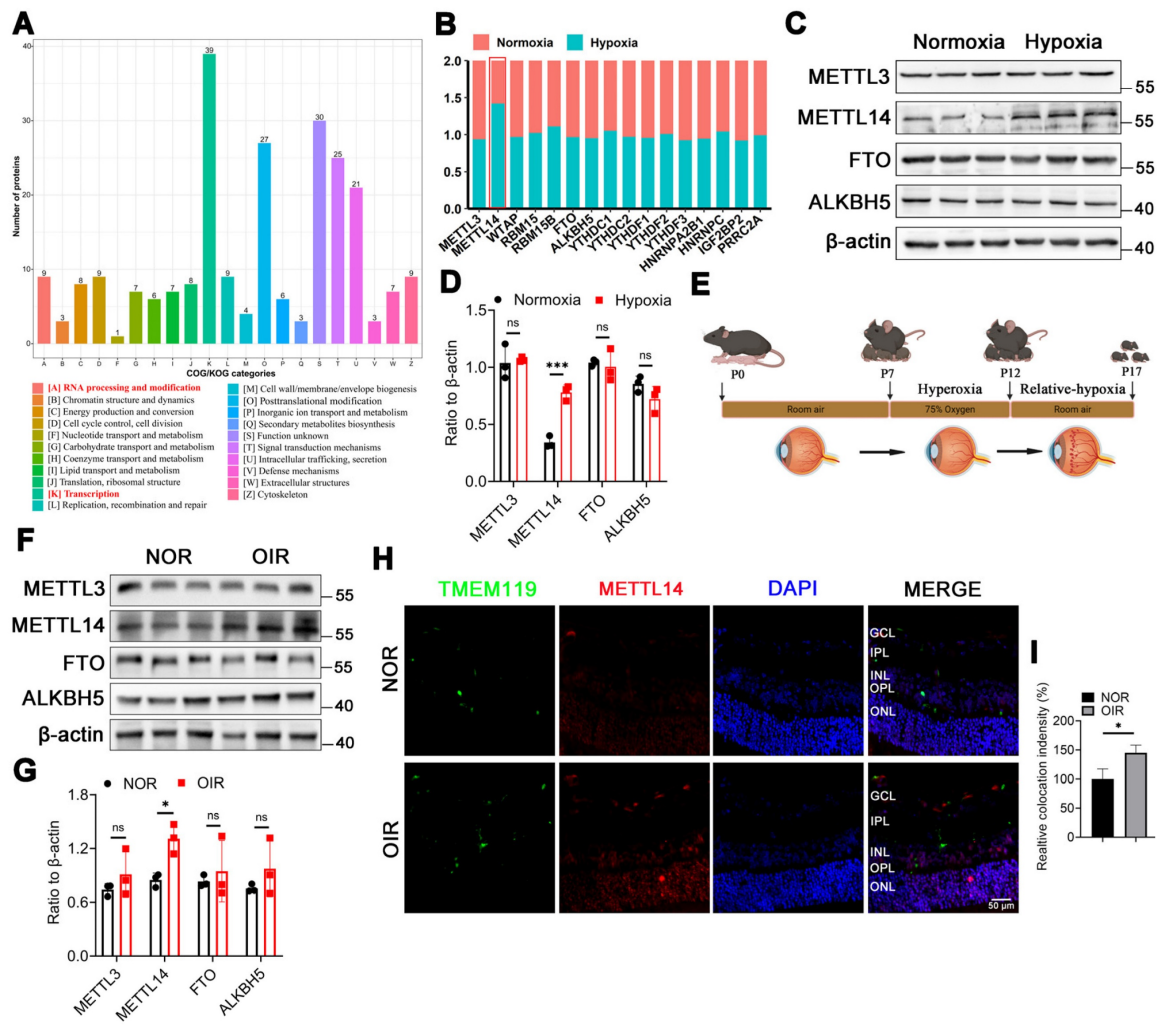
** METTL14 is significantly increased in hypoxic HMC3 cells and in OIR mice.** (A) COG analysis of proteomics on the proteomic data derived from HMC3 cells under normoxic and hypoxic conditions. (B) GO analysis of this proteomics. (C) Protein levels of m6A-related enzymes. (D, E) Protein bands and quantification of several crucial methylases, including METTL3, METTL14, FTO, ALKBH5 (mean ± SD; n = 3/group; ***P < 0.001, unpaired Student's t-test). (F) The modeling diagram of OIR mice. (G, H) The protein level and quantified chart of METTL14 in OIR mice (mean ± SD; n = 3/group; *P < 0.05, unpaired Student's t-test). (I) The immunofluorescence of METTL14 and TMEM119 (microglial marker) in retinal sections (Red, METTL14; Green, TMEM119; Blue, DAPI; Scale bar: 100 μm).

To investigate the crucial biological process involved in microglia-mediated angiogenesis, we revisited the proteomic dataset (PXD028737) from our previously published study on the hypoxic microglial cell line (HMC3). Clusters of Orthologous Groups (COG) analysis revealed a notable enrichment in transcription and RNA modification (Figure 1A). Given that N6-methyladenosine (m6A) methylation is the most prevalent eukaryotic mRNA base modification, playing a crucial role in mediating cellular signaling transduction, our focus was on m6A-related proteins. The proteomics showed that only METTL14 was up-regulated in hypoxic HMC3 cells (Figure 1B).

METTL3 is the core catalytic subunit of the m6A methyltransferase complex, responsible for adding m6A modifications to RNA. METTL14 is the auxiliary subunit of METTL3, forming a heterodimer with METTL3 to stabilize the complex structure and enhance its catalytic activity 26. FTO and ALKBH5 are the only two demethylases reported in the literature 27. Additionally, studies have reported that METTL3, METTL14, FTO, and ALKBH5 are associated with angiogenesis 28-30. Therefore, we selected these four proteins (METTL3, METTL14, FTO, and ALKBH5) to verify their roles in our study. The results suggested that the protein level of METTL14 was significantly increased in hypoxic HMC3 cells (Figure 1C-1D). Previous studies have emphasized the importance of m6A subcellular localization in its function 31, 32. Our results indicated that METTL14 maintained a consistent nuclear presence between normoxic and hypoxic HMC3 cells, with no significant changes in localization in response to hypoxia (Figure S1).

To validate the expression profile of these m6A-related proteins mentioned above in vivo, an oxygen-induced retinopathy (OIR) mouse model was established (Figure 1E). Western blot showed an increased level of METTL14 in the retinas of OIR mice compared with NOR mice (Figure 1F-1G). Our immunofluorescence analysis on retinal sections demonstrated a significant increase in the protein expression level of METTL14 within microglial cells in the retinas of OIR mice compared to normal mice (Figure 1H-1I). Overall, our findings implicated the m6A methyltransferase METTL14 contributes to the progression of retinal neovascularization.

### METTL14 directly binds to E3 ubiquitin-protein ligase BARD1

**Figure 2:**
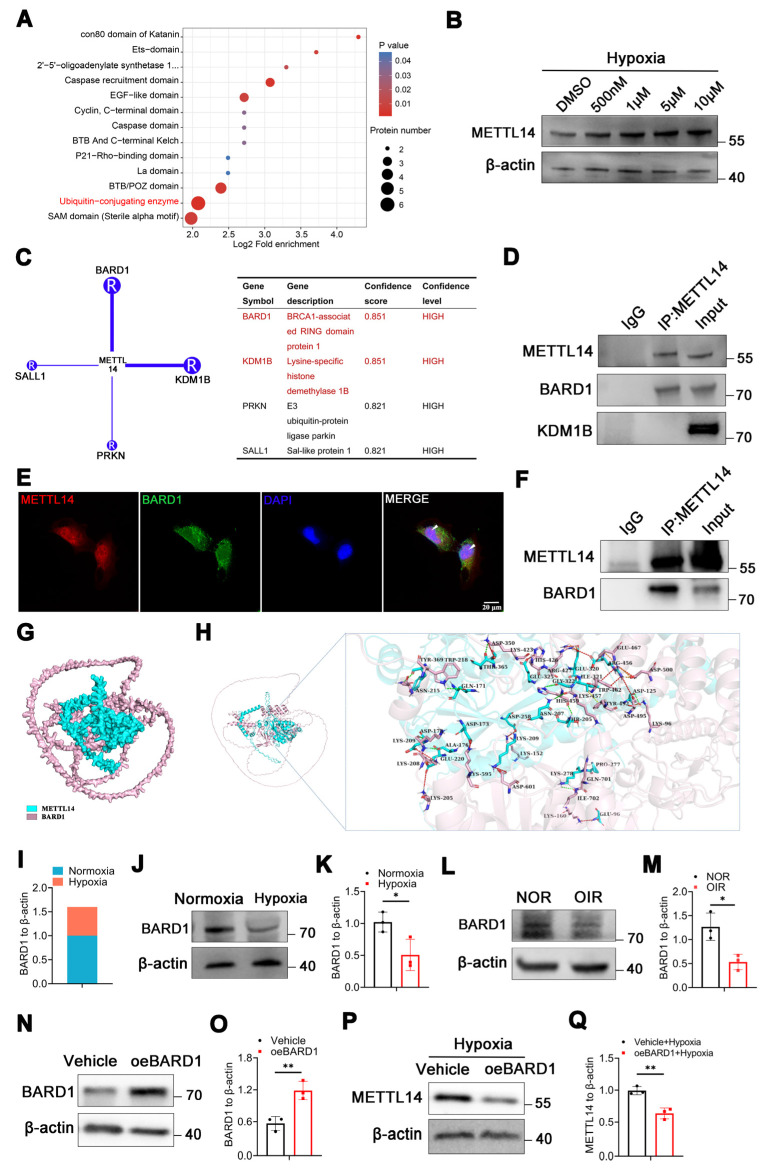
** METTL14 is regulated by E3 ubiquitin-protein ligase BARD1.** (A) Bioinformatic analysis of the proteomics data. (B) The protein level of METTL14 after treating with MG132 at 500 nM, 1 μM, 5 μM and 10 μM concentration. (C) Prediction of the ubiquitinase that interacts with METTL14. (D) Co-IP assay of METTL14, BARD1 and KDM1B in HMC3 cells. (E) The immunofluorescence of METTL14 and BARD1 in hypoxic HMC3 cells (Red, METTL14; Green, BARD1; Blue, DAPI; White arrow: co-location; Scale bar: 50 μm). (F) Co-IP experiment of METTL14 and BARD1 in the retinas of mice. (G, H) Analysis of the interaction between METTL14 protein and BARD1 protein. Green sticks represent the residues of the METTL14 protein, while light pink sticks depict the amino acid residues of the BARD1 protein. Dotted green lines: hydrogen bond interactions; Dotted light green line: C-H bond; Dotted orange line: salt bridge; Dotted red line: electrostatic interactions; Dotted yellow line: Pi-Sigma interaction. (I) The protein level of BARD1 from our proteomics data. (J, K) The protein level and quantification of BARD1 in normoxic and hypoxic HMC3 cells (mean ± SD; n = 3/group; *P < 0.05, unpaired Student's t-test). (L, M) The protein level and quantification of BARD1 in the retinas of NOR and OIR (mean ± SD; n = 3/group; *P < 0.05, unpaired Student's t-test). (N, O) The over-expression efficiency of BARD1 (mean ± SD; n = 3/group; **P < 0.01, unpaired Student's t-test). (P, Q) The protein level and quantification of METTL14 in hypoxic HMC3 cells with or without oeBARD1 (mean ± SD; n = 3/group; *P < 0.05, unpaired Student's t-test).

Intriguingly, the proteomics data revealed a significant enrichment of ubiquitin-conjugating enzymes (Figure 2A). Furthermore, the molecular function (MF) analysis also indicated a notable enrichment in the activities of ubiquitin-protein transferases and ubiquitin-like protein transferases (Figure S2A). Ubiquitination, serving as an efficient signaling mechanism for protein degradation, plays a crucial role in the stability, localization, activity, and involvement of proteins in various cellular processes 33. We sought to investigate whether up-regulated METTL14 is associated with ubiquitination in microglia under hypoxia. The concentration of MG132 was determined using cell viability assays with the CCK-8 kit, and the results indicated that concentrations of 500 nM, 1 μM, 5 μM, and 10 μM were appropriate (Figure S2B). Treatment of HMC3 cells with varying concentrations of proteasome inhibitor MG132 (500 nM, 1 μM, 5 μM, and 10 μM) under hypoxic conditions resulted in an increase in METTL14 levels (Figure 2B), suggesting that ubiquitination might regulates the stability and expression of METTL14. To further investigate which ubiquitin ligase mediated the ubiquitination of METTL14, we used the UbiBrowsea 2.0 website to predict potential candidates 34. The E3 ubiquitin-protein ligase BARD1 and lysine demethylase 1B (KDM1B) emerged with the highest enriched score (Figure 2C). Co-IP experiments showed a direct interaction between METTL14 and BARD1 in HMC3 cells (Figure 2D).

Immunofluorescence assay also revealed a co-localization between them (Figure 2E). In vivo, the Co-IP assay demonstrated the direct bind relationship between METTL14 and BARD1 (Figure 2F). Furthermore, AlphaFold3 showed that there are multiple interactions between METTL14 and BARD1 (Figure 2G-2H). Additionally, our proteomics data showed that BARD1 was decreased in HMC3 cells under hypoxia (Figure 2I). Then, we performed western blot and confirmed the down-regulated protein level of BARD1 in hypoxic HMC3 cells (Figure 2J-2K). In the retinas of OIR mice, we also observed a decrease protein level of BARD1 (Figure 2L-2M). Upon overexpressing BARD1 (oeBARD1) in HMC3 cells, western blot analysis indicated an approximately twofold increase in BARD1 protein levels (Figure 2N-2O). After overexpressing BARD1, the extent of METTL14's ubiquitination exhibited a significant increase (Figure S2C). Moreover, under hypoxic conditions, oeBARD1 in HMC3 cells exhibited a significant reduction in METTL14 protein levels (Figure 2P-2Q).

In summary, our results suggested that BARD1 can bind directly with METTL14, and under hypoxia, its decreased expression leads to the up-regulation METTL14 protein levels.

### Inhibition of METTL14 in microglia reduces cell adhesion

To assess the role of METTL14 in microglia, lentiviruses with METTL14 knockdown (sh-METTL14) were constructed. Fluorescent imaging was utilized to confirm the high efficiency of transfection (Figure S3A). The mRNA silencing efficiency achieved by sh-METTL14-2 and sh-METTL14-3 result in an approximate 70% reduction (Figure S3B), while the protein suppression extent of sh-METTL14-3 was approximately 80% (Figure S3C-S3D). Following comprehensive comparative analyses, HMC3 cells were transfected with the lentiviral sh-METTL14-3 for subsequent studies and were subjected to selection using puromycin dihydrochloride (2 μg/mL) to cultivate a stable transgenic line.

**Figure 3:**
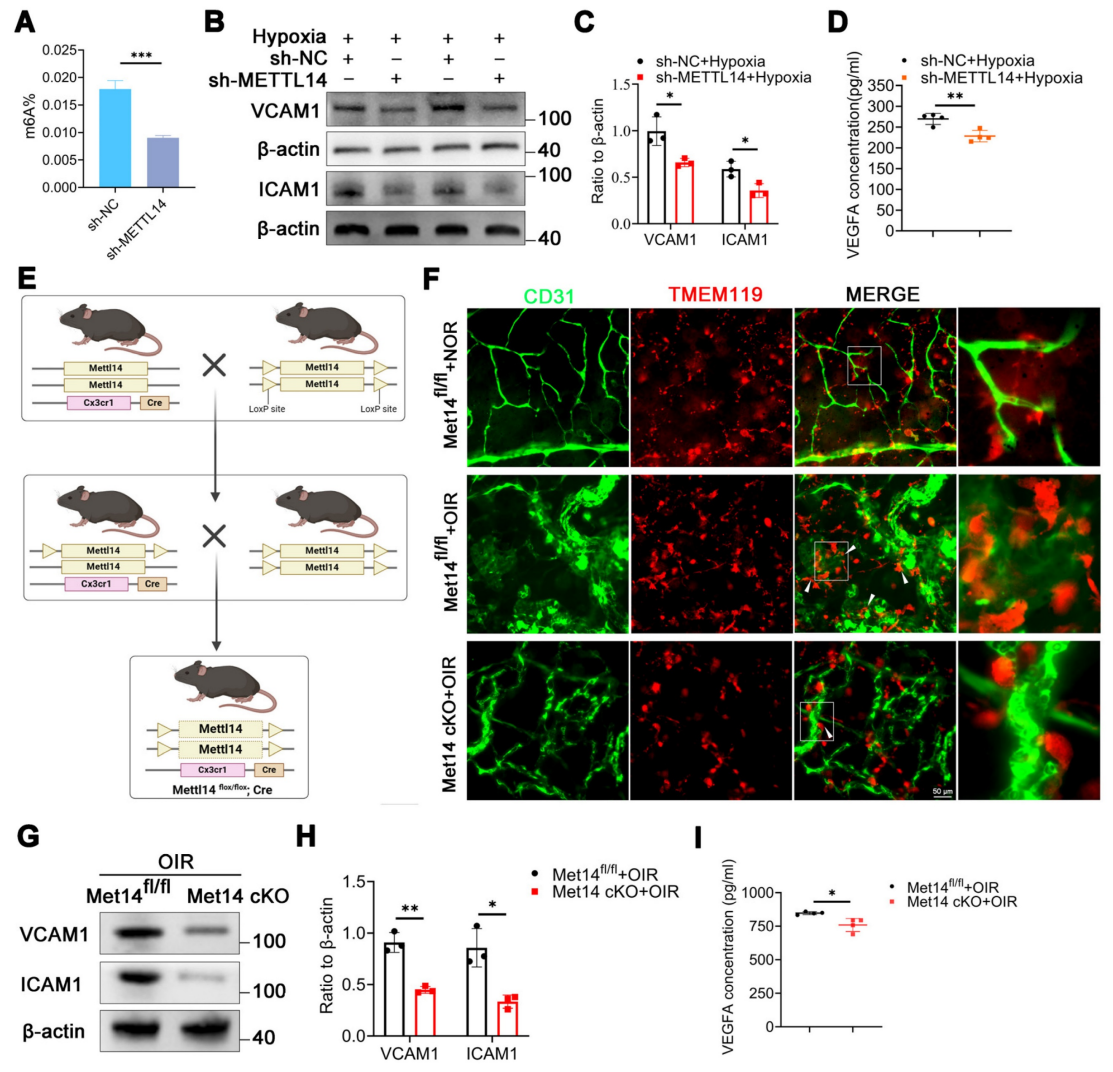
** The cell adhesion ability of microglia is reduced after silencing METTL14.** (A) The global m6A abundance in hypoxic HMC3 cells after inhibiting METTL14 (mean ± SD; n = 3/group; ***P < 0.001, unpaired Student's t-test). (B, C) VCAM1 and ICAM1 protein levels in HMC3 cells following with METTL14 knockdown under hypoxia conditions (mean ± SD; n = 3/group; *P < 0.05, unpaired Student's t-test). (D) The secretion of VEGFA in sh-NC and sh-METTL14 groups (mean ± SD; n = 4/group; *P < 0.05, unpaired Student's t-test). (E) The construction chart of Mettl14 cKO mouse. (F) The immunofluorescence of CD31 (Endothelial marker) and TMEM119 (Microglial marker) in retinal flat-mounts (White arrow, cell adhesion; Scale bar: 50 μm). (G, H) The protein levels and quantitative chart of VCAM1 and ICAM1 in the retinas of control and Mettl14 cKO mice with OIR (mean ± SD; n = 3/group; *P < 0.05, **P < 0.01, unpaired Student's t-test). (I) The secretion of VEGFA in the retinas of control and Mettl14 cKO mice under OIR conditions (mean ± SD; n = 4/group; *P < 0.05, unpaired Student's t-test).

Subsequently, the total m6A level was assessed in HMC3 cells with or without sh-METTL14 interference, revealing a significant reduction in the total m6A abundance in HMC3 cells following METTL14 knockdown (Figure 3A), suggesting a pivotal role for METTL14 in m6A modification within HMC3 cells.

Microglial polarization is associated with retinal vessel formation 35, 36. We investigated the expression of M1-type markers (iNOS, TNF-a) and M2-type markers (CD206, ARG1) in hypoxic HMC3 cells with sh-NC or sh-METTL14. The results showed that METTL14 did not influence microglial polarization (Figure S3E-S3H). It has been reported that a significant correlation exists between the adhesive capabilities of microglia and angiogenesis, a relationship that is especially critical in the context of retinal angiogenesis 37. Microglia exert their influence on angiogenesis through a spectrum of mechanisms, which encompass the secretion of various factors, modulation of gene expression, and direct engagement with endothelial cells 38, 39. Then, we examined two key adhesion molecules VCAM1 and ICAM1, and found that these two proteins were significantly decreased after knocking down METTL14 (Figure 3B-3C). ELISA assay showed that the secretion of VEGFA was reduced after silencing METTL14 in hypoxic HMC3 cells (Figure 3D).

Furthermore, we generated Mettl14 conditional knockout (Met14 cKO) mice by crossing the floxed Mettl14 mice with Cx3cr1-Cre (specifically expressed in microglia) transgenic mice (Figure 3E). Met14 cKO mouse was identified through DNA agarose gel spot electrophoresis experiment (Figure S3I). To exclude the possible effects of METTL14 conditional knockout on mice vision, we performed H&E staining and electroretinogram (ERG). The results showed that it did not influence retinal structure and retinal function (Figure S3J-S3P).

After establishing the OIR model, retinal flat-mount was staining with CD31 (endothelial marker) and TMEM119 (microglial marker). The findings revealed that under normoxic conditions, pathological neovascularization ensued, characterized by the presence of convoluted vessels and an accompanying increase in permeability, microglia were uniformly dispersed with characteristic ramified morphology. However, upon exposure to hypoxic conditions, numerous microglia migrated towards areas of neovascularization, closely adhering to neovascular clusters and adopting an amoeboid shape. In contrast, following the conditional knockout of METTL14, a decreased cell adhesion capacity and a reduced number were observed as compared with the control group (Figure 3F, Figure S3Q). Furthermore, we found that the protein levels of VCAM1 and ICAM1 were significantly decreased in the retinas of Met14 cKO mice with OIR as compared with control group (Figure 3G-3H). The ELISA assay also revealed a decrease in the secretion of VEGFA in the retinas of Met14 cKO mice with OIR (Figure 3I).

### Silencing METTL14 alleviates retinal neovascularization

**Figure 4:**
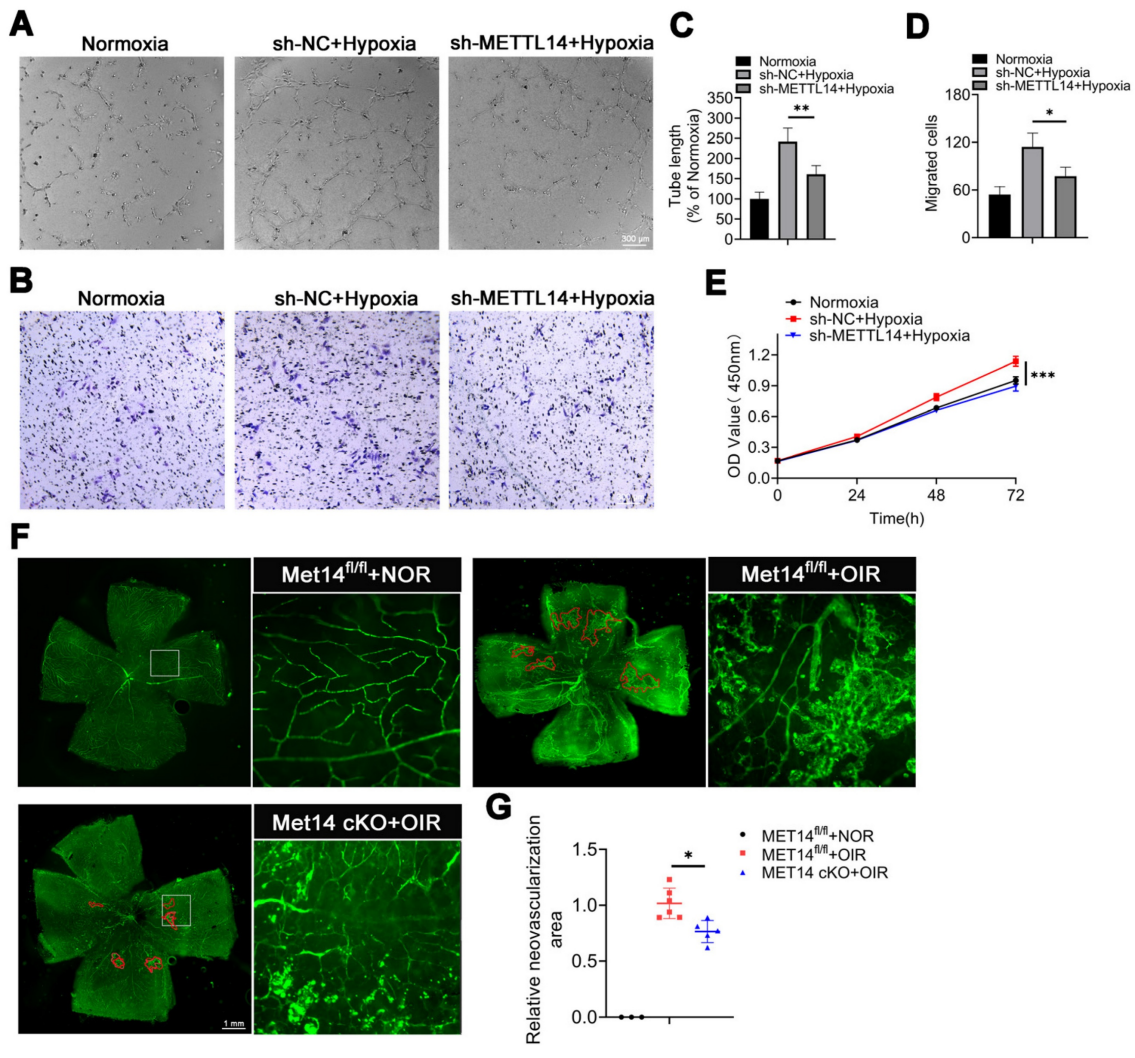
** The angiogenesis-related abilities of HRMECs are diminished after coculturing with hypoxic sh-METTL14 HMC3 cells.** (A, C) Tube formation ability and quantification of HRMECs that co-cultured with normoxic, hypoxic sh-NC, and hypoxic sh-METTL14 HMC3 cell (mean ± SD; n = 4/group; **P < 0.01, One-way ANOVA; Scale bar: 300 μm). (B, D) Migrated ability and quantitative graph of HRMECs that co-cultured with above three groups (mean ± SD; n = 4/group; *P < 0.05, One-way ANOVA; Scale bar: 300 μm). (E) Proliferative ability of HRMECs that co-cultured with three groups mentioned above (mean ± SD; n = 5/group; ***P < 0.001, One-way ANOVA). (F, G) Immunofluorescence of CD31 in retinal flat-mounts and quantified graph (mean ± SD; n ≥ 5/group; **P < 0.01, One-way ANOVA).

To explore the role of METTL14 in the process of angiogenesis, we devised a co-culture system to examine how HMC3 cells influence HRMECs (Human retinal microvascular endothelial cells) (Figure S4). A series of angiogenesis-related functional tests were performed, the results showed that tube formation, migration and proliferation of HRMECs were enhanced when co-cultured with hypoxic HMC3 cells compared to normoxic condition. Additionally, these abilities were diminished upon suppression of microglial METTL14 in the co-culture system (Figure 4A-4E). Similarly, in vivo experiments revealed a large number of neovascular clusters in peripheral retina with OIR; however, conditional knockout METTL14 in microglia attenuated angiogenesis area (Figure 4F-4G).

Collectively, these findings imply that METTL14 facilitates retinal angiogenesis. Moreover, silencing of METTL14 in microglia appears to significantly impede vessel formation.

### The transcription factor MXD1 is directly modified by METTL14

**Figure 5:**
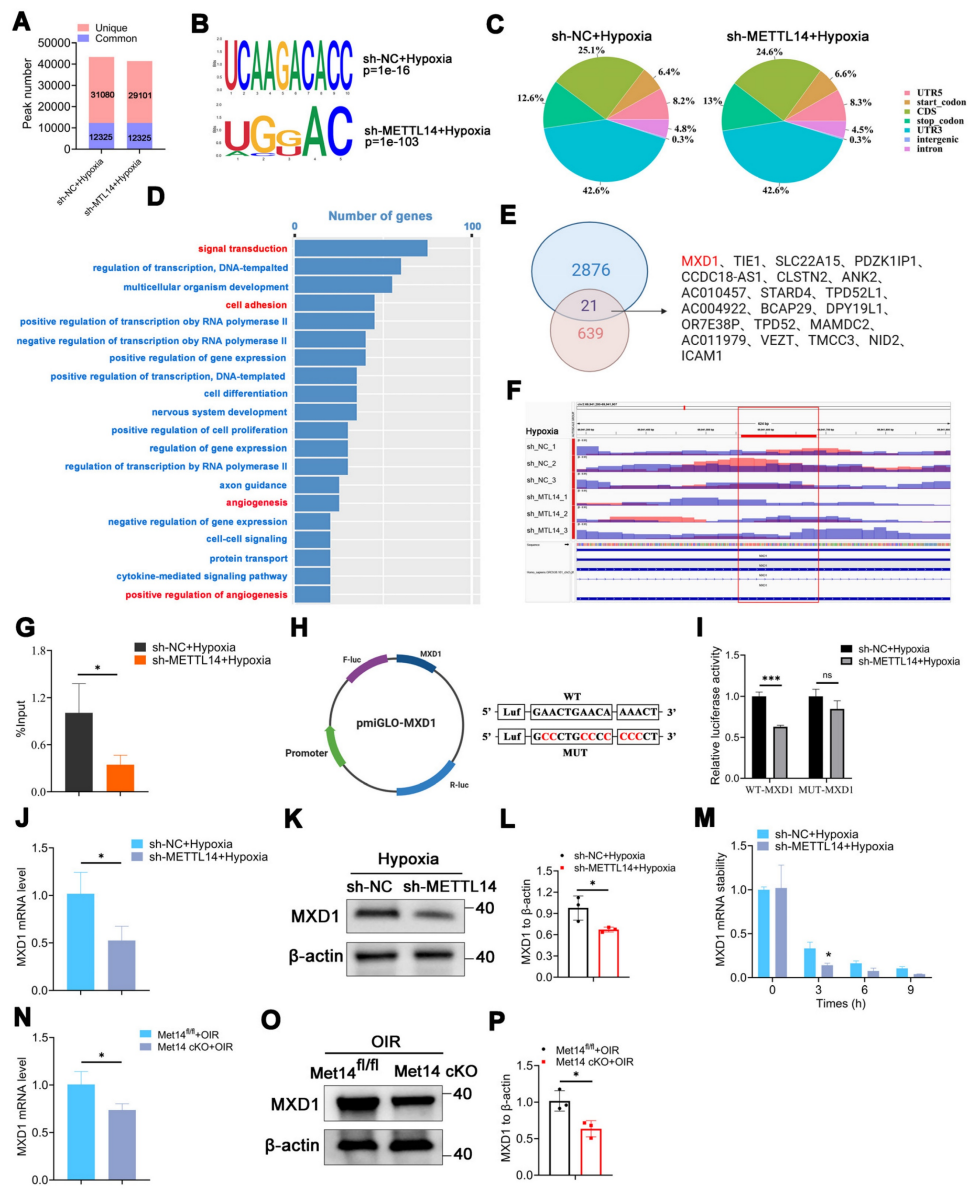
** Identification of MXD1 through integrated analysis of MeRIP-seq and RNA-seq.** (A) Common and unique peak numbers in hypoxic HMC3 cells with or without sh-METTL14. (B) m6A motif in sh-NC or sh-METTL14 HMC3 cells with hypoxia. (C) m6A peak distribution in 5'UTR, start codon, CDS, stop codon, 3'UTR, intergenic, and intron regions. (D) GO analysis of hypoxic sh-NC and sh-METTL14 HMC3 cells. (E) Intersection genes identified in both MeRIP-seq and RNA-seq datasets. (F) m6A abundance of MXD1 between sh-NC and sh-METTL14 groups with hypoxia. (G) MeRIP-qPCR of MXD1 in the two groups mentioned above (mean ± SD; n = 3/group; *P < 0.05, unpaired Student's t-test). (H) Construction of dual luciferase carrier and sequence of MXD1. (I) Relative dual-luciferase activity of MXD1 in HMC3 cells with sh-NC and sh-MET14 under hypoxic conditions (mean ± SD; n = 3/group; ***P < 0.001, unpaired Student's t-test). (J) MXD1 mRNA level following METTL14 inhibition (mean ± SD; n = 3/group; *P < 0.05, unpaired Student's t-test). (K, L) Protein level and quantification of MXD1 in HMC3 cells with or without sh-METTL14 (mean ± SD; n = 3/group; *P < 0.05, unpaired Student's ttest). (M) MXD1 mRNA ability in the two groups following Act-D treatment (mean ± SD; n = 3/group; *P < 0.05, unpaired Student's t-test). (N) MXD1 mRNA level in the retinas of control and Mettl14 cKO mice with OIR (mean ± SD; n = 3/group; *P < 0.05, unpaired Student's t-test). (O, P) Protein level and quantification of MXD1 in the retinas of control and Mettl14 cKO mice under OIR conditions (mean ± SD; n = 3/group; *P < 0.05, unpaired Student's t-test).

To explore the underlying mechanism, we conducted methylated RNA immunoprecipitation sequencing (MeRIP-seq) and RNA-sequencing across sh-NC or sh-METTL14 groups subjected to hypoxic condition, ensuring robust analysis supported by three independent biological replicates. The data revealed that the quality thresholds were met robustly, with Q20 exceeding 95%, Q30 surpassing 90%, and a GC content greater than 50%, indicating high-quality sequencing control (Table S1). MeRIP-seq identified 43405 and 41426 peaks in sh-NC or sh-METTL14-HMC3 cells with hypoxia, respectively, with 12325 common peaks of m6A modification determined in both groups (Figure 5A). Additionally, the m6A consensus motif was highly enriched with RRACH (R= A/G, H=A, C/ U) (Figure 5B). The peak density of m6A-modification was especially enriched in the stop codon or 3′-untranslated region (UTR) in both groups (Figure 5C), consistent with previous study 40. Further investigate of the m6A peak distribution indicated a similar pattern (Figure S5).

GO analysis suggested that METTL14 was closely related with signal transduction, cell adhesion, and angiogenesis (Figure 5D). Combined analysis with MeRIP-seq and RNA-seq pinpointed 21 significantly down-regulated genes. Within this subset, attention was particularly drawn to transcription factors (TFs). Remarkably, among the 21 genes, only one transcription factor was identified-MAX dimerization protein 1 (MXD1) (Figure 5E). MXD1 plays a role in regulating cell proliferation and chemokines 41, 42, both of which are necessary for angiogenesis. However, its specific function in hypoxic microglia still require further research. Integrative genomics viewer (IGV) revealed that the m6A modification of MXD1 was significant reduced after knocking down METTL14 (Figure 5F). To verify the directly modified relationship, MeRIP with an m6A-specific antibody followed by RT-qPCR revealed that the m6A modification level on MXD1 mRNA was indeed significantly decreased (Figure 5G). Furthermore, to pinpoint the modification site, we engineered pmirGLO-MXD1 luciferase reporter plasmids containing the 3'UTR sequence of MXD1 along with a mutant variant (Mut-3'UTR) of this sequence. The results showed that METTL14 significantly enhanced the expression of MXD1 3'UTR reporter in WT, not mutated (Figure 5H-5I), suggesting m6A-dependent regulation of RNA stability. Then, we validated the decreased mRNA expression of MXD1 in sh-METTL14 group (Figure 5J), consistent with the RNA-seq data. Correspondingly, a decreasing trend was also observed at the protein level (Figure 5K-5L). Moreover, sh-NC and sh-METTL14-HMC3 cells were treated with Act-D (5 μg/mL) to block transcription. The results showed that the mRNA stability of MXD1 was reduced in METTL14 silenced cells with hypoxia (Figure 5M). It indicated that m6A modification influenced the degradation of MXD1. Furthermore, in vivo experiments revealed that the mRNA level of MXD1 was reduced in the retinas of Mettl14 cKO mice with OIR (Figure 5N). Western blot also showed a decrease protein of MXD1 in the retinas of Mettl14 cKO mice under OIR conditions (Figure 5O-5P).

Our data delineated the landscape of m6A modifications in hypoxic HMC3 cells with or without METTL14 knockdown, and identified the downstream target gene MXD1.

### Overexpressing MXD1 enhances vessel formation-related abilities in METTL14-knockdown HMC3 cells

**Figure 6:**
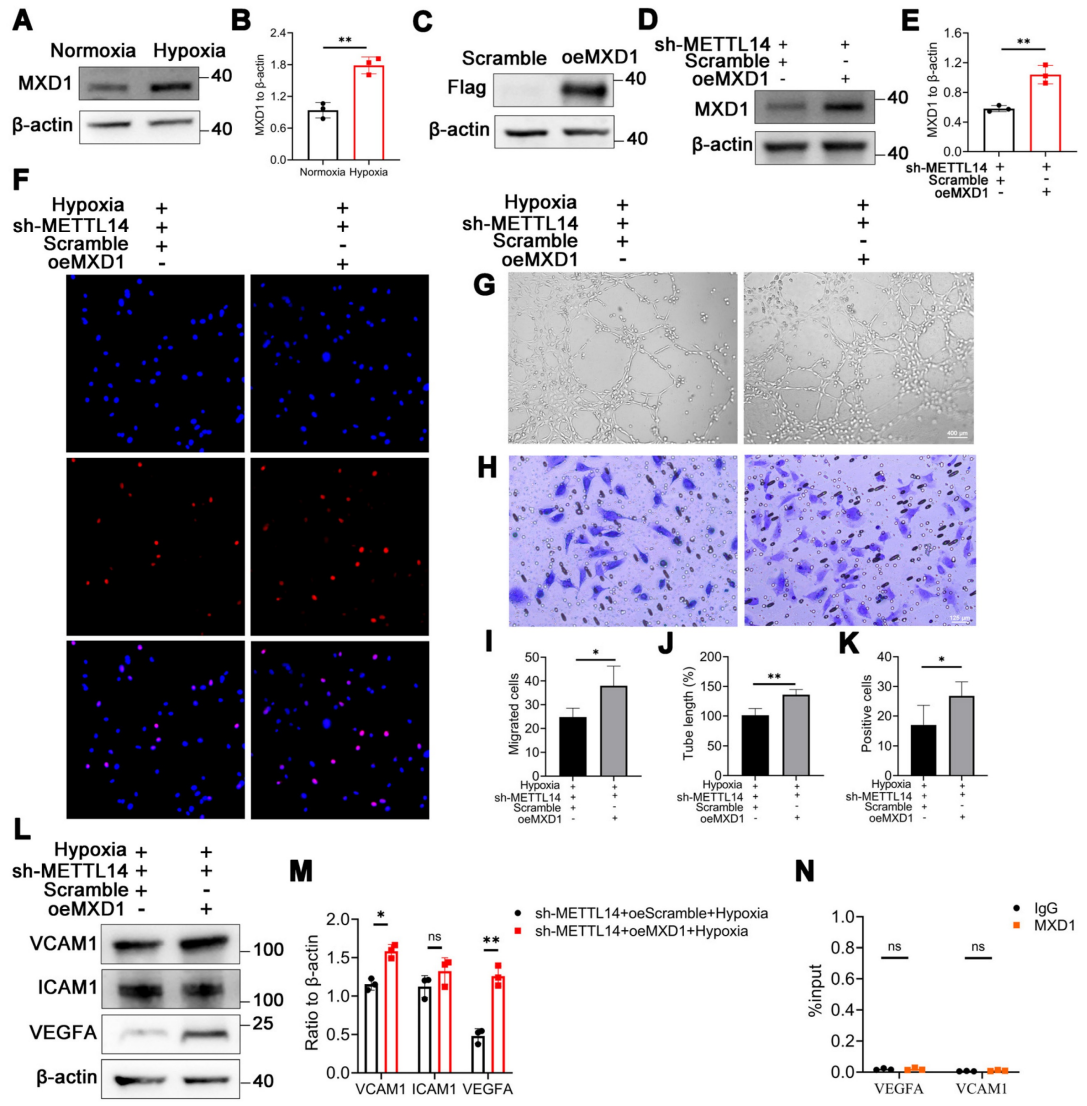
** The angiogenesis-related capabilities of HRMECs are increased in oeMXD1 group.** (A, B) MXD1 protein level in normoxic and hypoxic HMC3 cells (mean ± SD; n = 3/group; *P < 0.05, unpaired Student's t-test). (C) The protein bands of Flag in HMC3 cells with or without oeMXD1. (D, E) The overexpressing efficiency of MXD1 (mean ± SD; n = 3/group; **P < 0.01, unpaired Student's t-test). (F, I) The proliferative ability of HRMECs that co-cultured with sh-METTL14 HMC3 cell with Scramble or oeMXD1 (mean ± SD; n = 3/group; *P < 0.05, unpaired Student's t-test). (G, J) The tube formation ability of HRMECs in the groups mentioned above (mean ± SD; n = 4/group; **P < 0.01, unpaired Student's t-test). (H, K) The migrated capability of HRMECs that co-culture with the two groups (mean ± SD; n = 4/group; *P < 0.05, unpaired Student's t-test). (L, M) The protein levels and quantification of VCAM1, ICAM1, and VEGFA in sh-METTL14-HMC3 cells with Scramble or oeMXD1 under hypoxia (mean ± SD; n = 3/group; *P < 0.05, **P < 0.01, unpaired Student's t-test). (N) ChIP-qPCR of VEGFA and VCAM1 following with MXD1 immunoprecipitation (mean ± SD; n = 3/group; ns > 0.05; unpaired Student's t test).

We noted a significant increase in the protein level of MXD1 in HMC3 cells under hypoxia (Figure 6A-6B), suggesting the potential pathogenic role of MXD1. Subsequently, we constructed lentiviruses overexpressing MXD1 with FLAG-tag, and infected sh-METTL14-HMC3 cells with them. The FLAG-tag was detected in the MXD1 overexpression group, with no corresponding band observed in the Scramble control group (Figure 6C). Western blot analysis revealed that MXD1 overexpression led to an approximate two-fold increase in MXD1 protein levels (Figure 6D-6E). Following this, we performed a series of functional assays related to vessel formation. The results demonstrated that the tube formation, migratory and proliferative capacities of HRMECs were enhanced when co-cultured with overexpressed MXD1 group compared to Scramble group (Figure 6F-6K). Western blot showed that the protein levels of VCAM1 and VEGFA were significantly up-regulated after overexpressing MXD1 in hypoxic sh-METTL14 HMC3 cells (Figure 6L-6M). We remain uncertain about the mechanisms by which MXD1 regulates the expression of VEGFA and VCAM1. Considering the well-established role of MXD1 as a transcription factor, we conducted ChIP-qPCR assays to investigate whether MXD1 directly binds to the promoters of VEGFA or VCAM1. However, the results indicated that TF-MXD1 does not directly bind to the promoters of these genes (Figure 6N). This suggests that MXD1 may regulate the expression of these genes through alternative, indirect mechanisms. Further studies are needed to elucidate these potential regulatory pathways.

### MXD1 is recognized and regulated by m6A reader YTHDF2

**Figure 7:**
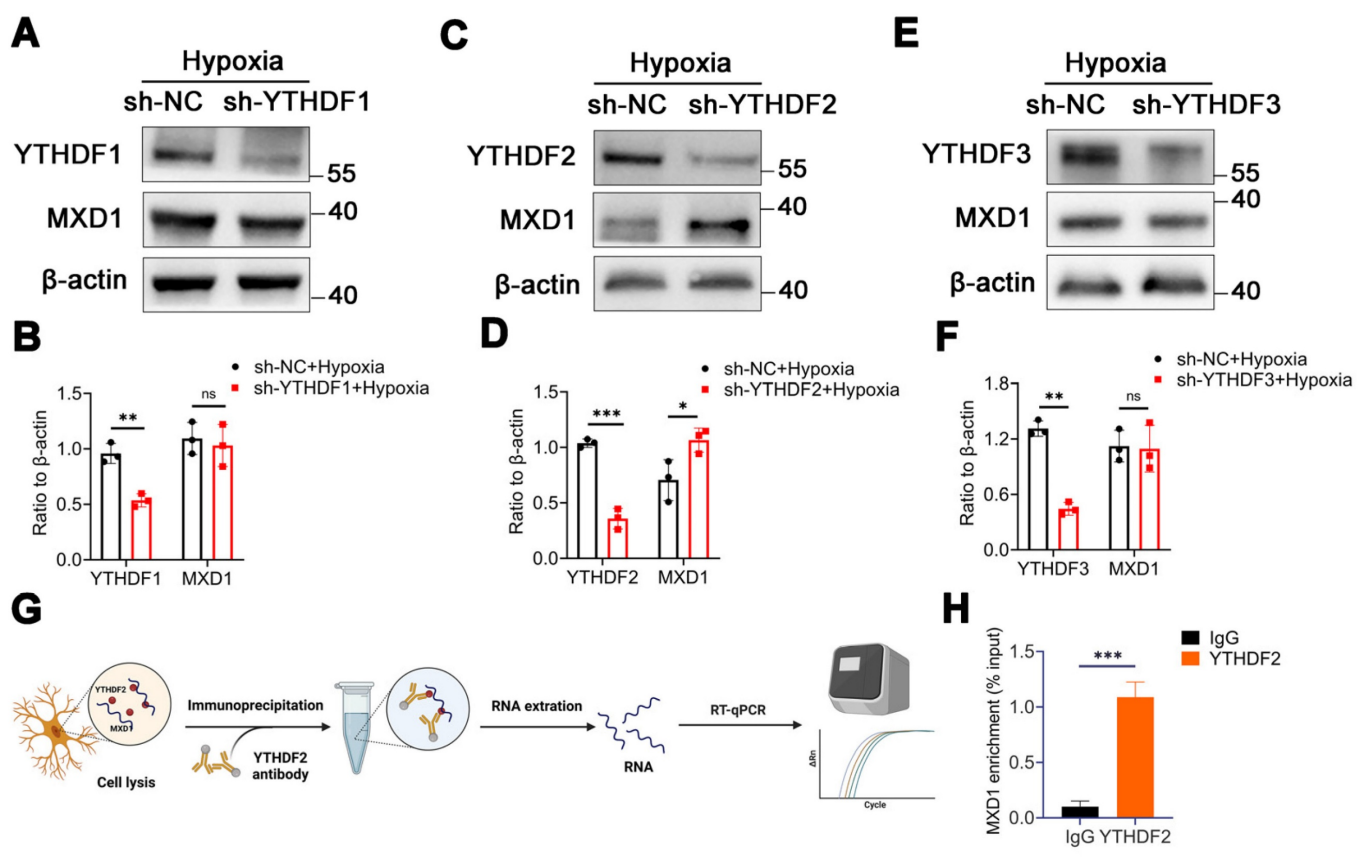
** MXD1 is recognized by YTHDF2.** (A-F) The protein level of MXD1 following with YTHDF1/2/3 knockdown (mean ± SD; n = 3/group; ns >0.05, **P < 0.01, ***P < 0.001, unpaired Student's t-test). (G) The chart of RIP-qPCR experiment. (H) RIP-qPCR of MXD1 following with YTHDF2 immunoprecipitation (mean ± SD; n = 3/group; ***P < 0.001, unpaired Student's t-test).

**Figure 8:**
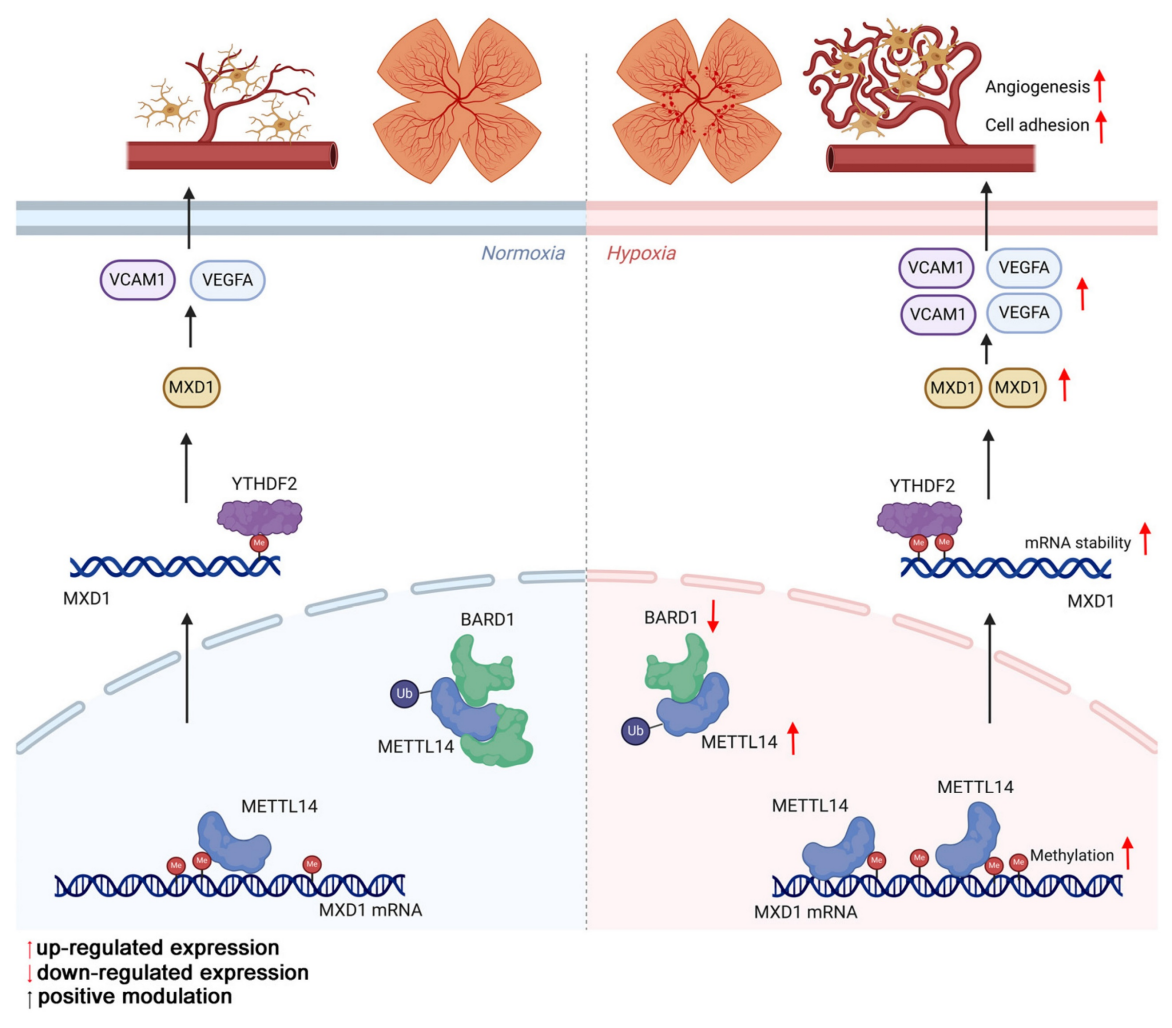
The mechanism by which METTL14 regulates retinal angiogenesis.

In our investigation, we discovered evidence suggesting that the stability of MXD1 mRNA is influenced due to m6A modification. The YTH domain-containing family protein encompasses YTHDF1/2/3, which can recognize and bind to mRNA containing m6A modifications, directing them to cellular degradation pathways like RNA fragmentation or processing bodies 43, 44. Therefore, we hypothesis that MXD1 might be recognized by YTH family proteins. Lentiviruses carrying YTHDF1/2/3 were constructed and used to infect HMC3 cells. Following exposure to hypoxia, a markedly reduced MXD1 level was observed in the sh-YTHDF2 group (Figure 7A-7F), suggesting regulation of MXD1 by YTHDF2. Moreover, to establish the relationship between MXD1 and YTHDF2, the RNA immunoprecipitation (RIP) assay was conducted (Figure 7G). The results revealed a direct binding of YTHDF2 to MXD1 mRNA (Figure 7H). Taken together, our findings suggested that ubiquitination of METTL14 promotes retinal neovascularization via m6A-modification on MXD1 mRNA in oxygen induced retinopathy (Figure 8).

## Discussion

Retinal vascular diseases, which include conditions such as retinopathy of prematurity (ROP), diabetic retinopathy (DR), and age-related macular degeneration (AMD), are characterized by the formation of irregular and leaky microvessels 45-50. Throughout this pathological progression, there is heightened activity of retinal microglia, the primary immune guardians of the central nervous system crucial for maintaining retinal homeostasis 8, 51. However, in the diseased state, these cells may secrete various pro-inflammatory cytokines and angiogenic growth factors, further intensifying disease advancement 52-54. However, the pathological mechanisms involved are still poorly understood.

The m6A modification represents a prevalent epigenetic modification on mRNAs, integral to modulating numerous essential cellular processes 55. Recent studies unveiled that m6A methylation alterations are crucial for microglia plasticity 56. Within the central nervous system, microglia serve as pivotal agents for immune surveillance and orchestrating inflammatory responses. The m6A modifications within these glial cells can influence the synthesis of various inflammatory mediators, modulating the inflammatory cascade 23, 57. Furthermore, m6A modification acts as a fundamental regulator of microglial stress response, enabling these cells to adapt to environmental changes and execute reparative functions effectively. In our study, the proteomics showed an enrichment of the biological process in transcription and RNA modification in hypoxic HMC3 cells. Among various RNA modifications, N6-methyladenosine (m6A) is the most abundant and well-studied form 58. Given its prevalence and biological significance, we therefore specifically focused on m6A in this study. Moreover, western blot and immunofluorescence revealed that the expression of methylase METTL14 was upregulated. Thus, we speculated that METTL14-mediated microglia plasticity might be closely related to the progression of retinal neovascularization.

METTL14, an integral constituent of the m6A methylation machinery, acts in concert with METTL3 as an RNA m6A methyltransferase, primarily tasked with the incorporation of the m6A modification marker onto mRNA 55, 59. Such modifications via METTL14 can significantly alter RNA stability, translation efficacy, and processing dynamics, thus governing cellular destiny and functionality, with profound implications for disease pathogenesis 60-62. Li et al. suggested that METTL14 regulates microglia/macrophage polarization and NLRP3 inflammasome activation via the KAT3B-STING axis following ischemic stroke 24. This finding highlights the potential role of METTL14 in modulating the inflammatory response of microglia. Moreover, it is reported that silencing METTL14 could reduce primary microglial proliferation and pyroptosis by stabilizing HDAC3 expression 63. Collectively, these studies suggest that METTL14 has multiple functions in regulating the behavior of microglia.

While m6A modifications are omnipresent across diverse cellular contexts, their influence on biological functions and disease manifestation subtly shifts based on the unique microenvironment and the cellular condition. However, it remains unclear what leads to the high expression of METTL14. Through the UbiBrowser website 34, 64, we found that E3 ubiquitin-protein ligase BARD1 may directly bind to METTL14, leading to an increased METTL14 levels, confirmed by Co-IP experiments in vitro and in vivo. To investigate the effect of METTL14 on retinal neovascularization, in vitro experiments were performed, showing that inhibition of METTL14 in HMC3 cells notably attenuated angiogenic capabilities in co-cultured endothelial cells. Moreover, we constructed a conditional knockout of METTL14 and observed that targeted suppression of METTL14 within the microglia markedly diminished pathological angiogenesis.

To elucidate the underlying molecular mechanisms, we utilized MeRIP-seq and RNA-seq, which uncovered that METTL14 can directly target MXD1 mRNA to modify it. RT-qPCR demonstrated that METTL14 could regulate the expression of MXD1. Subsequently, MeRIP-qPCR and Act-D assays confirmed the m6A modification on MXD1 and its regulation of mRNA stability, thereby further substantiating the pivotal role of m6A modification in regulating MXD1. MXD1 encodes Max Dimerization Protein 1, a key transcription factor regulating gene expression by binding to specific DNA sequences 65. This protein plays a crucial role in cellular processes like proliferation, differentiation, migration, and apoptosis 66, 67, and is implicated in various diseases, notably cancer 68, 69. In our study, predictive algorithms and RIP assays identified that YTHDF2 directly interacts with m6A-modified MXD1 mRNA, modulating its expression levels. Furthermore, our results demonstrated that MXD1 overexpression in METTL14-deficient HMC3 cells restored angiogenic tube formation capacities of HRMECs, highlighting the critical role of MXD1 in angiogenesis. Prior studies have recognized METTL14 as a pivotal factor in m6A modification, Zhang et al. 21 discovered that the Mettl14 mRNA expression increased in a hypoxia/reoxygenation model. Knocking down Mettl14 significantly curbed the proliferation of endogenous neural stem cells. Similarly, another study focused on hypoxia-regulated ferroptosis in HCC cells and pinpointed the HIF-1α/METTL14/YTHDF2/SLC7A11 axis as a potential therapeutic target for HCC interventional embolization treatment 70. Our research not only highlights the closely relationship of m6A in OIR but also specific the pro-angiogenesis function of METTL14. Moreover, this study delineates METTL14 upregulation mechanism under hypoxic conditions and its direct impact on MXD1 mRNA stability and expression. This insight introduces a novel perspective on how hypoxia modulates gene expression via RNA modifications. Collectively, these studies, including ours, highlight the multifaceted roles of METTL14 in various cellular contexts under hypoxic conditions.

In reflecting upon our research, it is important to acknowledge certain limitations. While we have established, through in vivo studies with mice and cellular analyses, that elevated expression of METTL14 is associated with increased angiogenesis, we have yet to corroborate these findings with clinical patient samples. Our future endeavors will involve collecting and examining of specimens from patients to further strengthen our hypotheses. Additionally, since m6A modifications can variably influence different cell types and tissues 71, our study was constrained by the challenge of acquiring a sufficient quantity of primary microglia from the retina and their isolation from astrocytes. Consequently, we utilized the HMC3 microglial cell line. Moving forward, we are committed to devising strategies to procure and isolate primary microglia to deepen our research. In addition, to explore whether effects of METTL14 on microglia depend on its demethylation activity, we plan to construct inactive METTL14 plasmids to further explore the role of METTL14's methyltransferase activity in the future.

In conclusion, our study reveals that METTL14 in microglia promotes pathological retinal neovascularization through binding with MXD1 mRNA in a YTHDF2-dependent manner, offering new therapeutic strategies and potential targets for RNV diseases including ROP.

## Material and methods

### Cell culture and reagents

The human microglial cell line HMC3 was acquired from the American Type Culture Collection (ATCC). The human retinal microvascular endothelial cell (HRMEC) was obtained from BLUEFBIO, Shanghai, China. The HMC3 cells were cultivated in MEM-F12 medium (BasalMedia, Shanghai) supplemented with 10% fetal bovine serum (FBS, Gibco, USA), while the HRMEC cells were grown in DMEM-F12 medium (Procell Life Science&Technology Co.,Ltd) with the same supplement. Cells exposed to a condition of 21% oxygen were termed normoxia, whereas those subjected to 2% oxygen were defined as hypoxic exposure in vitro. Serum-free cell freezing medium was purchased from Witcel (A401, Shanghai). MG132 was purchased from TargetMol (T2154, USA). Actinomycin D was purchased from MedChemExpress (HY-17559, Shanghai).

### Oxygen-induced retinopathy (OIR) Model

Briefly, breastfeeding female mice were housed with postnatal day 7 (P7) newborn mice in an oxygen chamber (Changsha Huaxi Electronics Technetronic Co.,ltd) with hyperoxia (75%±1%O_2_) for 5 d. At P12, the mice were removed from the oxygen chamber and continued to be housed in a normal oxygen environment (~21%O_2_) for an additional 5 d. The control group mice were raised in a normal oxygen environment all the time. The Ethics Committee of the First Affiliated Hospital of Chongqing Medical University (2021-612) gave its approval to each study protocol involving the use of animals.

### Cell Counting Kit-8 (CCK-8) assay

HMC3 cells were processed using Trypsin-EDTA solution (T1300, Solarbio) for dissociation and then seeded at a density of 5 x 10^3^ cells per well into 96-well plates. Cell quantification was performed using a cell counter (Countstar, China). Once the cells had adhered to the plate surface, the existing culture medium was aspirated. Subsequently, 110 µL of fresh medium containing 10 µl of the Cell Count Kit-8 (C0005, TargetMol, USA) was added to each well, and the mixture was incubated for an additional 2.5 h. The absorbance of the samples was then determined at a wavelength of 450 nm using a microplate reader (Thermo Fisher Scientific, Inc., MA, USA).

### Enzyme-linked immunosorbent assay (Elisa)

The supernatant of HMC3 cells with different treatment and retinas of Mettl14 cKO mice with OIR were collected. Subsequently, the concentration of Vascular Endothelial Growth Factor A (VEGFA) was quantitatively determined using ELISA kits (Elabscience, Wuhan, China), following the manufacturer's recommended protocols. The microplate reader (Thermo Fisher Scientific, Inc., MA, USA) was employed to measure the absorbance values at a wavelength of 450 nm.

### mRNA stability assay

sh-NC and sh-METTL14 knockdown HMC3 cells were treated with 5 μg/mL actinomycin D (HY-17559, MCE, Shanghai) for 0, 3, 6, and 9 h under hypoxia. At each time point, cells were harvested and subjected to RNA extraction. RT-qPCR was employed to assess the mRNA levels of MXD1 across all groups.

### Dual-luciferase reporter assay

A luciferase assay was conducted using reporter lysis buffer (E1910, Promega, USA) according to the manufacturer's guidelines. In brief, sh-NC and sh-METTL14 cells were transfected with wild-type MXD1 and mutant MXD1 plasmids (GeneCreate, Wuhan) into a 24-well plate (Jet Biofil). After a 24 h incubation, the cells were assayed using the Dual-Glo Luciferase Assay System (Promega). Renilla Luciferase (R-luc) was utilized to normalize the Firefly Luciferase (F-luc) activity, thereby assessing the efficiency of reporter translation.

### Real-Time Quantitative PCR (RT-qPCR)

RNA isolation was carried out using TRIzol Reagent (Thermo Fisher Scientific) as directed by the manufacturer's guidelines. cDNA synthesis was achieved with the RT Master Mix (AG11705, Accurate Biotechnology (Hunan) Co., Ltd, ChangSha, China). The qPCR mix was consisted with free water, primer and SYBR Green Real-Time PCR Master Mixes (AG11708, Accurate Biotechnology (Hunan) Co., Ltd, ChangSha, China). Then, the qPCR mix was dispensed into a 96-well PCR plate (A-GEN) and subjected to amplification and detection using the ABI 7500 Real-Time PCR System (Applied Biosystems, USA). β-actin was employed as the housekeeping gene. Relative gene expression was determined using the 2^-ΔΔCt^ approach. The primers were applied in Table S2.

### ChIP-qPCR

The ChIP assay was performed using the Chromatin Immunoprecipitation Kit (17-295, Millipore, Germany) according to the manufacturer's instructions. Briefly, HMC3 cells were cross-linked using 1% formaldehyde (12606S, Cell Signaling Technology) for 15 mins at room temperature, followed by quenching with a concentration of 0.125 M glycine for 10 mins. Cells was then washed with PBS once, and resuspend in lysis buffer and incubate on ice for 10 mins. Cross-linked chromatin was sheared using sonication (5% energy, 25 s for 8 cycles). The sheared chromatin was incubated with beads coated with 6 mg of anti-MXD1 antibody (19547-1-AP, Proteintech) or IgG control at 4°C overnight. The immunoprecipitated DNA was purified and subjected to qPCR analysis.

### Western blot

Proteins were extracted from tissue and cells using RIPA Buffer (R0020, Solarbio). Samples were separated by SDS-PAGE employing 4%-20% polyacrylamide gels (ET15420Gel, ACE Biotechnology). After electrophoresis, the protein samples were transferred to a 0.45 μm polyvinylidene difluoride (PVDF) membrane (Millipore, IPVH00010) and subsequently blocked with Fast Blocking Western reagent (Yeasen, Shanghai). The membranes were then incubated with primary antibodies at 4°C for over 16 h, then washed with TBST for three times, followed by a 1 h incubation with secondary antibodies at room temperature. The signals were detected using an ECL kit (4AW011-100, 4A Biotech) and quantified with ImageJ software, with the results normalized to β-actin expression levels. The antibodies used in this study were provided in Table S3.

### Lentivirus transfection

HMC3 cells were seeded at a density of 2 x 10^5^ cells per well into 6-well plates. After the cells had adhered to the wall, lentiviruses were transfected into the 6-well plates as directed by the manufacturer's guidelines. Then, the cells were cultured in medium containing 2 μg/mL puromycin to obtain stably infected cells. Lentiviruses for MXD1 overexpression were obtained from OBiO Technology (Shanghai) Co., Ltd. The tag antibody Flag was obtained from GenScript Corporation. METTL14 knockdown lentivirus was designed by Shanghai Genechem Co., Ltd, while YTHDF1-3 knockdown lentiviruses were developed by Chengdu Yueyong Dajiang Technology Co., Ltd.

### Methylated RNA immunoprecipitation sequencing (MeRIP-seq)

Briefly, mRNA was purified from total RNA samples and chemically digested into fragments of 100 nucleotides in length. MeRIP was conducted to selectively enrich m6A-methylated mRNAs using an anti-m6A antibody (202003, Synaptic Systems). Both m6A-enriched RNAs and input mRNAs were prepared for library construction using the TruSeq Stranded mRNA Library Preparation kit (Illumina). The libraries were then denatured to produce single-stranded DNA molecules, which were subsequently captured on Illumina flow cells and amplified in situ to generate sequencing clusters. These clusters were sequenced for 150 cycles on an Illumina HiSeq 4000 system, following the manufacturer's protocol. ExomePeak was employed for peak calling, and significant MeRIP-enriched regions (peaks) were identified for each transcript and compared using exomePeak. These regions (peaks) were annotated using the most up-to-date Ensembl database to assign genes. Statistical analysis was conducted to compare the m6A peaks within each transcript region. MeRIP-seq was replicated three times for each condition to ensure biological variability was accounted for.

### RNA-sequencing

Total RNA was extracted from HMC3 cells using Trizol reagent (Thermo Fisher Scientific), as previously described. For RNA-sequencing, the RNAs were subjected to single-end sequencing on Illumina HiSeq 2000 platforms at Lc-Bio Technology Co., Ltd. Three separate biological replicates were conducted for each group in the RNA-seq experiment.

### RNA immunoprecipitation (RIP)

The RIP assay was conducted in accordance with the manufacturer's instructions (Bes5101, BersinBio, Guangzhou). In essence, HMC3 cells were lysed using a polysome lysis buffer to release RNA and proteins from the cytoplasm. A total of 10 µg of the target antibody YTHDF2 (ab220163, Abcam) or IgG was added to Protein A/G agarose beads to prepare the bead-antibody complex. Then, 100 µL of the supernatant was aspirated and combined with 900 µL of the bead-antibody complex, followed by an overnight rotation incubation at 4°C. Subsequent to several washing steps, any unbound RNA and proteins were removed. The RNA within the immunoprecipitated complex was extracted and subjected to RT-qPCR to quantify the abundance of MXD1 in the pellet.

### Co-immunoprecipitation (Co-IP)

HMC3 cells were cultured according to the method described above. Co immunoprecipitation (Co-IP) was performed using Thermo Scientific Pierce Co-IP kit (Thermo Fisher Scientific, USA) according to the manual instruction. Samples were analyzed by Western blotting using anti-IgG (30000-0-AP, Proteintech), anti-METTL14 (A24396, Abclonal), anti-BARD1 (A1685, Abclonal).

### m6A RNA methylation quantification

RNA extraction was performed as previously described. To assess the global levels of m6A RNA methylation in mRNA, an EpiQuik m6A RNA Methylation Quantification Kit (P-9005-96, Epigentek, USA) was employed according to the manufacturer's instructions. For each sample, 200 ng of mRNA was used. Absorbance readings were taken at a detection wavelength of 450 nm.

### 5-ethynyl-2´-deoxyuridine (Edu) staining

HMC3 cells were cultured with or without hypoxic conditions for 24 h and then transferred to the upper chambers (0.4 μm; Corning, Inc.) at a density of 5×10^4^ cells per well. Meantime, HRMECs were seeded into the basolateral chambers at the same density. After 24 h of co-culture, the upper chambers were removed, and HRMECs were incubated with 1 mL of 1x EdU solution (10 μM, Beyotime, Shanghai) for 30 mins. Subsequently, cellular fixation was performed using a 4% paraformaldehyde fixative for 10 mins, followed by permeabilization with 0.3% Triton X-100 (BL935A, Biosharp) for an additional 30 mins and blocked by 3% goat serum (MB4510, MeilunBio). The cells were then exposed to a 200 μL reaction mixture from the EdU kit for 30 mins and finally stained with 1x Hoechst 33342 (23491-52-3, Cytoch) for 5 mins. Imaging was carried out using a fluorescence microscope (Leica, Germany).

### Tube formation assay

The initial cells were handled as previously described. Following this, HRMECs were digested and seeded into 96-well plates (BDBIO HangZhou China) containing Matrigel (356234, Corning) at a density of 1×10^4^ cells per well. After a 6 h incubation period, images were captured using a microscope (Leica, Germany).

### Transwell assay

HMC3 cells were cultured under normoxic or hypoxic conditions for 24 h and then transferred to the lower chambers (8 μm, NEST Biotechnology) at a density of 2×10^4^ cells per well. Meantime, HRMECs were seeded into the upper chambers at the same density. After 24 h of co-culture, the chambers cultured with HRMECs were taken out, HRMECs were immobilized using a 4% Paraformaldehyde Fix Solution (BL539A, Biosharp) for 15 mins, followed by three washes. The cells were then stained with 1% crystal violet (C0121, Beyotime). A cotton swab was used to gently remove cells from the upper surface of the chamber membrane, and images of the migrated cells on the lower surface were captured using a microscope (Leica, Germany).

### Methylated RNA immunoprecipitation qPCR (MeRIP-qPCR)

The procedure of m6A immunoprecipitation (MeRIP) was performed on the basis of previously reported methods 72. The RNA fragments of sh-NC and sh-METTL14 groups were generated and then the fragmented RNA samples were divided into two parts: one part was used for immunoprecipitation (IP) experiments and served as the IP sample after IP, while the other part was not subjected to IP and was directly used as the Input sample. Equal volumes of RNA were subjected to reverse transcription and followed by qPCR validation. The expression level of genes in the IP groups relative to the Input groups was expressed as a percentage of the Input.

### Immunofluorescence staining and retinal flat-mount

The pre-stage of embedding of eyeball was performed by Wuhan servicebio technology Co.,Ltd. Retinal section was dewaxed by xylene and absolute ethyl alcohol sequentially, then the tissue section was placed in a repair box filled with EDTA antigen repair buffer for antigen repair in a microwave oven. Blocked by goat serum for 30 mins, followed with incubation of METTL14 antibody (NBP1-81392, Novusbio) and TMEM119 antibody (ab209064, Abcam) at 4℃ overnight. Washed with PBS for three times, the section was incubated with Cy3-labeled Goat Anti-Rabbit (A0516, Beyotime) and Alexa Fluor 488-labeled Goat Anti-Mouse (A0428, Beyotime) at room temperature for 1 h. Finally, the section was sealed with anti-fluorescence quenching tablets containing DAPI solution (KGE2505-1, Keygen BioTECH). Images were captured by Fluorescence microscopy (Leica, Germany).

Mice were euthanized, then the eyes were taken out and fixed in 4% paraformaldehyde at room temperature for approximately 2 h. The retinas were gently dissected into a quadrifoliate configuration and mounted on a glass slide under microscope (Olympus, Japan). After permeabilizing the cell membranes, the antigens were blocked with goat serum for 30 mins at 37°C. The sealing solution was carefully removed, and PBS containing a specific ratio of CD31 antibody (ab182981, Abcam) was added to the slices. The slices were then placed horizontally in a humidified box and incubated overnight at 4°C. Fluorescence microscopy (Leica, Germany) was used to capture images.

### Mettl14 conditional knockout (cKO) mice

Mettl14^f/f^ mice were kindly provided by the Southwest Hospital of AMU. Cx3cr1-Cre mice were built by Cyagen Biosciences Inc. These mice were then bred to produce microglia-specific homozygous (Mettl14^f/f^; Cx3cr1) and control mice. DNA segment was synthesised using the 2×Taq PCR Mix (PMT-01, ProMab Biotechnologies Inc.). The PCR products obtained, using 5'loxp-F and 5'loxp-R primers were separated using agar gel electrophoresis. A 380-bp band was detected as Cre-Cx3cr1. A 545 bp band was detected the loxp sequence, the primer sequence for Mettl14 was: F: CTGCCTGAACCTCTTGAGAACTGA; R: GCAGACAAGTGAGGAAATAAGCAAG. The primer sequence for Cx3cr1 was: GACATTTGCCTTGCTGGAC; R: GCAGGGAAATCTGATGCAAG. All mice were maintained on a C57BL/6J background and housed in a specific pathogen-free facility under a 12 h light/dark cycle at a temperature of 24 ± 2°C and a humidity ranging from 30% to 70%.

### Statistical analysis

In SPSS analysis, two-tailed Student's t-tests were employed for comparing two groups, and one-way ANOVAs were utilized for comparing three groups. A P<0.05 was considered statistically significant to determine the differences. All data sets are presented as means ± standard deviations.

## Supplementary Material

Supplementary figures and tables.
