# Supplementary material for: BARD1-mediated stabilization of METTL14 promotes retinal neovascularization by m6A-modifying MXD1 mRNA on a YTHDF2-dependent manner
Source: Theranostics. 2025 Apr 13;15(11):5481–98. doi: 10.7150/thno.110122 (PMC12036877; doi:10.7150/thno.110122)
Supplement: Supplementary file 1 — Supplementary figures and tables. [file thnov15p5481s1.pdf]

Supplementary Table. 1. Data quality control.

Supplementary Table. 2. The Primers for RT-qPCR used in this study.

Supplementary Table. 3. The Antibodies for western blotting used in this study.

Supplementary Figure. 1. The co-location between METTL14 and DPAI.

Supplementary Figure. 2. (A) The molecular function of the proteomics. (B) The cell viability (mean ± SD; n= 5/group; *P < 0.05, One-way ANOVA). (C) The ubiquitination of METTL14.

Supplementary Figure. 3. (A) The transduction efficiency of METTL14 lentivirus. (B) The mRNA knockdown efficiency of METTL14 (mean ± SD; n= 3/group; ***P < 0.001, One-way ANOVA). (C, D) The protein knockdown level of METTL14 (mean ± SD; n= 3/group; ***P < 0.001, One-way ANOVA). (E-H) The marker of M1-type and M2-type (mean ± SD; n= 3/group; ns > 0.05, unpaired Student's t-test). (I) Sample 2 (S2), S3, S4, S6, S7, S9, S10 were identified as homozygous Mettl14$^{fl/fl}$ mice, while S2, S5, S6, S7, S8 carried the Cx3cr1-Cre transgene. In summary, S2, S6, S7 were identified as homozygous Mettl14 cKO mice. (J-O) A wave and B wave of ERG between WT and Mettl14 cKO mice. (P) The H&E staining of WT and Mettl14 cKO mice. (Q) The immunofluorescent staining of TMEM119 and CD31 in OIR mice.

Supplementary Figure. 4. The co-cultured system.

Supplementary Figure. 5. The m6A peak distribution.

Supplementary Table. 1 Data quality control

Samples	**Q20%**	Q30%	GC%
Sh-NC-1-IP	98.17	94.62	51.14
Sh-NC-2-IP	98.13	94.50	50.91
Sh-NC-3-IP	98.19	94.68	51.08
Sh-MET14-1-IP	98.20	94.72	51.81
Sh-MET14-2-IP	98.11	94.47	50.95
Sh-MET14-3-IP	98.14	94.51	51.13
Sh-NC-1-input	98.36	94.96	49.18
Sh-NC-2-input	98.29	94.80	49.18
Sh-NC-3-input	98.36	94.98	49.29
Sh-MET14-1-input	98.37	95.03	49.93
Sh-MET14-2-input	98.31	94.82	49.39
Sh-MET14-3-input	98.31	94.81	49.46

Supplementary Table. 2 The Primers for RT-qPCR used in this study

Genes	Species	Sequences (5'-3')
METTL3	Human	CATTGCCCACTGATGCTGTG
		AGGCTTTCTACCCCATCTTGA
METTL14	Human	AGTGCCGACAGCATTGGTG
		GGAGCAGAGGTATCATAGGAAGC
FTO	Human	GCTGCTTATTTCGGGACCTG
		AGCCTGGATTACCAATGAGGA
ALKBH5	Human	CGGCGAAGGCTACACTTACG
		CCACCAGCTTTTGGATCACCA
MXD1	Human	TTTCCCTGTCAACCTAAG
		GAAATAAGCCCACAAAAT
β-ACTIN	Human	CATGTACGTTGCTATCCAGGC
		CTCCTTAATGTCACGCACGAT
Mxd1	Mouse	AGATGCCTTCAAACGGAGGAA
		CAAGCTCAGAGTGGTGTGTCG
β-actin	Mouse	GGCTGTATTCCCCTCCATCG
		CCAGTTGGTAACAATGCCATGT

Supplementary Table. 3 The Antibodies for western blotting used in this study

Antibody	Host	Manufacturer	Application
β-actin	Mouse	Proteintech	1:10,000
METTL3	Rabbit	Proteintech	1:1,000
METTL14	Rabbit	NovusBio/Abclonal	1:1,000/1:50
FTO	Rabbit	Abcam	1:2,000
ALKBH5	Rabbit	Abcam	1:1,000
Ubiquitination	Rabbit	HuaBio	1:1,000
BARD1	Rabbit	Abclonal	1:1,000
Flag	Mouse	Origene	1:1,000
iNOS	Rabbit	Abcam	1:1,000
TNF-α	Rabbit	Proteintech	1:1,000
CD206	Rabbit	Proteintech	1:1,000
ARG1	Rabbit	Proteintech	1:1,000
VCAM1	Rabbit	Proteintech	1:1,000
ICAM1	Rabbit	Proteintech	1:1,000
YTHDF1	Rabbit	Proteintech	1:1,000
YTHDF2	Rabbit	Proteintech	1:1,000
YTHDF3	Rabbit	Proteintech	1:1000
MXD1	Rabbit	Affinity	1:1,000
VEGFA	Rabbit	Abcam	1:800

Fig .S1

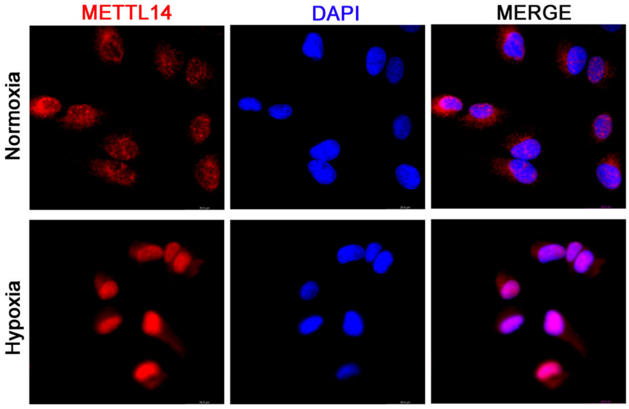

Fig. S2

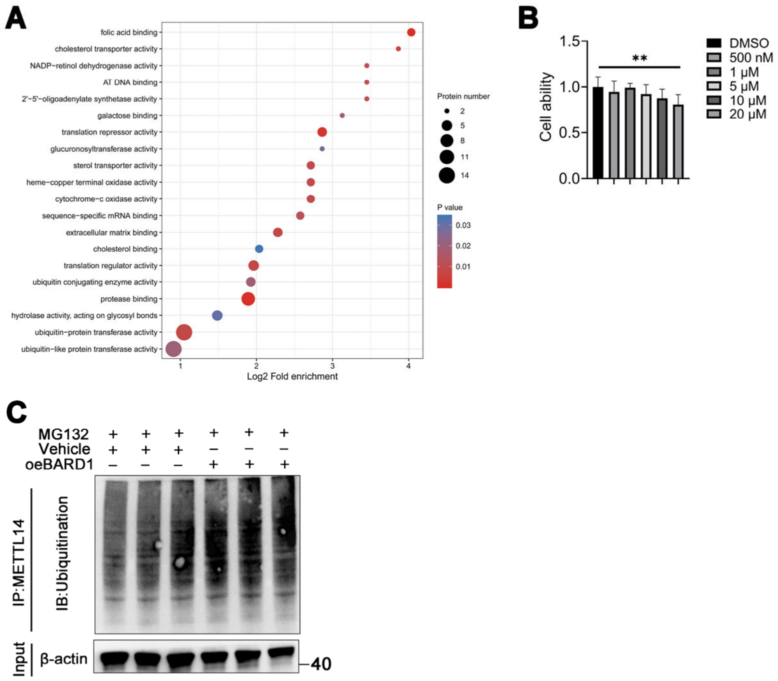
A
folic acid binding
cholesterol transporter activity
NADP−retinol dehydrogenase activity
AT DNA binding
2'−5'−oligoadenylate synthetase activity
galactose binding
translation repressor activity
glucuronosyltransferase activity
sterol transporter activity
heme−copper terminal oxidase activity
cytochrome−c oxidase activity
sequence−specific mRNA binding
extracellular matrix binding
cholesterol binding
translation regulator activity
ubiquitin conjugating enzyme activity
protease binding
hydrolase activity, acting on glycosyl bonds
ubiquitin−protein transferase activity
ubiquitin−like protein transferase activity
Log2 Fold enrichment
Protein number
2
5
8
11
14
P value
0.03
0.02
0.01
B
Cell ability
1.5
1.0
0.5
0.0
**
DMSO
500 nM
1 μM
5 μM
10 μM
20 μM
C
MG132
Vehicle
oeBARD1
IP:METTL14
IB:Ubiquitination
Input
β-actin
40

Fig. S3

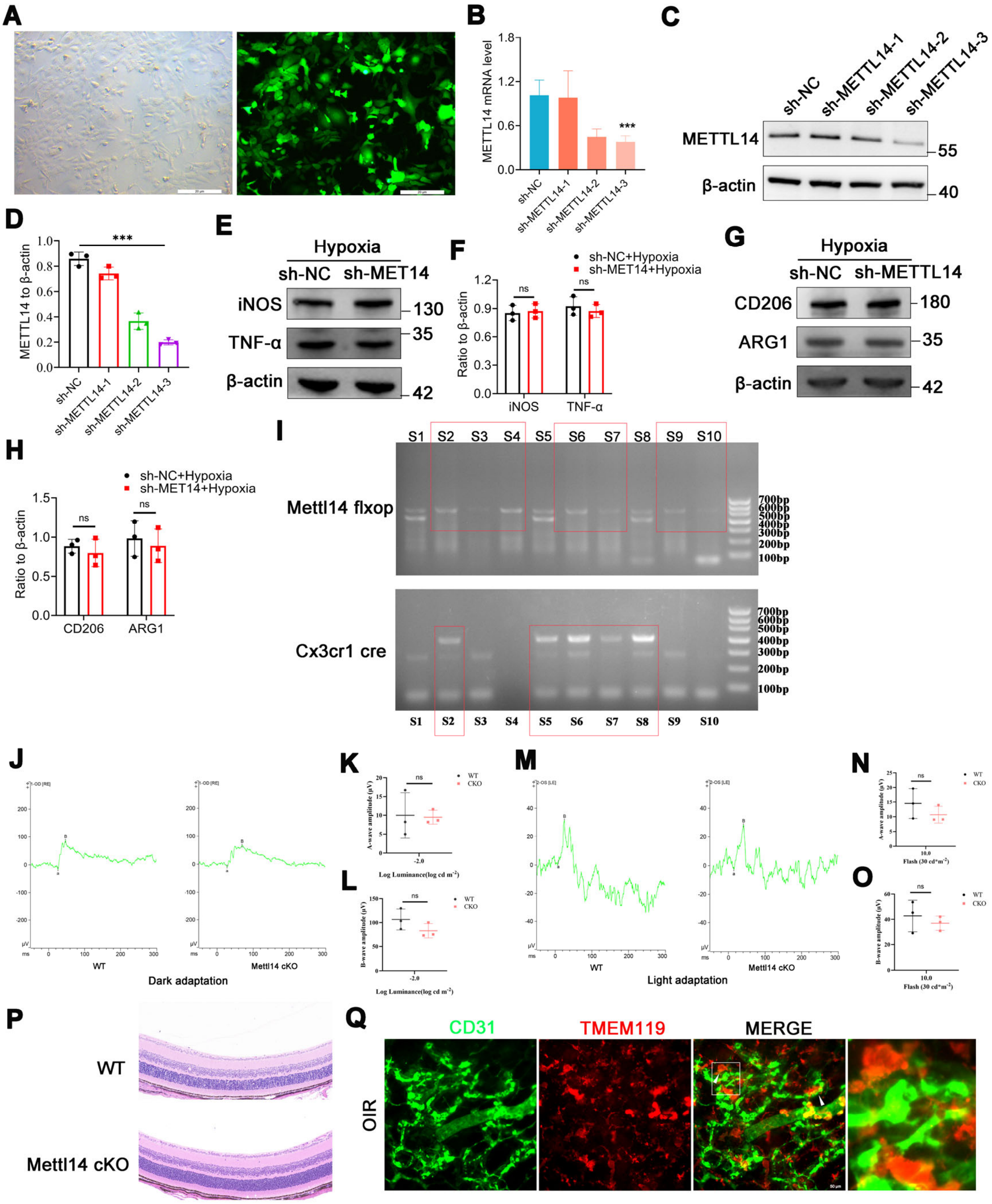

A
B
METTL14 mRNA level
sh-NC
sh-METTL14-1
sh-METTL14-2
sh-METTL14-3
***
C
METTL14
β-actin
55
40
D
METTL14 to β-actin
***
E
Hypoxia
sh-NC
sh-MET14
iNOS
TNF-α
β-actin
130
35
42
F
sh-NC+Hypoxia
sh-MET14+Hypoxia
Ratio to β-actin
ns
G
sh-METTL14
CD206
ARG1
180
H
I
S1 S2 S3 S4 S5 S6 S7 S8 S9 S10
Mettl14 flxop
Cx3cr1 cre
700bp
600bp
500bp
400bp
300bp
200bp
100bp
J
WT
Mettl14 cKO
Dark adaptation
K
A-wave amplitude (μV)
Log Luminance(log cd m⁻²)
WT
CKO
L
B-wave amplitude (μV)
M
Light adaptation
N
Flash (30 cd*m⁻²)
O
P
Q
CD31
TMEM119
MERGE
OIR

Fig. S4

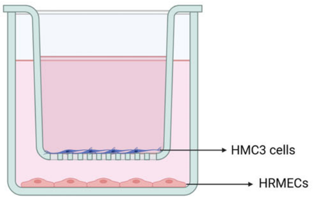

Fig. S5

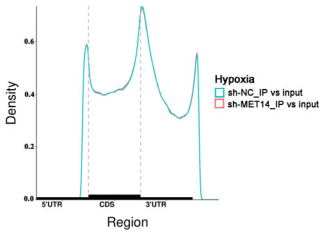
0.6
0.4
0.2
0.0
Density
5'UTR
CDS
3'UTR
Region
Hypoxia
sh-NC_IP vs input
sh-MET14_IP vs input
